# *Helicobacter pylori* VacA induces apoptosis by accumulation of connexin 43 in autophagic vesicles via a Rac1/ERK-dependent pathway

**DOI:** 10.1038/cddiscovery.2015.35

**Published:** 2015-09-28

**Authors:** K Yahiro, Y Akazawa, M Nakano, H Suzuki, J Hisatune, H Isomoto, J Sap, M Noda, J Moss, T Hirayama

**Affiliations:** 1 Department of Molecular Infectiology, Graduate School of Medicine, Chiba University, Chiba 260-8670, Japan; 2 Department of Gastroenterology and Hepatology, Nagasaki University Hospital, 1-7-1 Sakamoto, Nagasaki, Japan; 3 Department of Bacteriology, Institute of Tropical Medicine, Nagasaki University, Nagasaki 852-8523, Japan; 4 Division of Gastroenterology and Hepatology, Department of Internal Medicine, Keio University School of Medicine, 35 Shinanomachi, Shinjuku-ku, Tokyo 160-8582, Japan; 5 Department of Bacteriology, Hiroshima University Graduate School of Biomedical Sciences, Hiroshima 734-8551, Japan; 6 Division of Medicine and Clinical Science, Department of Multidisciplinary Internal Medicine, Tottori University School of Medicine, Tottori 683-8503, Japan; 7 University Paris Diderot, Sorbonne Paris Cité, Epigenetics and Cell Fate, UMR 7216 CNRS, Paris, France; 8 Cardiovascular and Pulmonary Branch, NHLBI, National Institutes of Health, Bethesda, Maryland 20892, USA

## Abstract

*Helicobacter pylori* (*H. pylori*) produces vacuolating cytotoxin (VacA), a potent protein toxin, which is associated with gastric inflammation and ulceration. Recent studies demonstrated that connexins (Cxs), which are responsible for intracellular communication at gap junctions (GJs) as well as cell homeostasis, participate in VacA-induced cell death. We now demonstrate in AZ-521 cells that VacA increased cytoplasmic Cx43, accompanied by LC3-II generation in a time- and dose-dependent manner without induction of Cx43 mRNA expression. Inhibition of VacA-induced Rac1 activity prevented ERK phosphorylation and the increase in Cx43. Suppression of ERK activity and addition of *N*-acetyl-cysteine inhibited VacA-dependent increase in Cx43 and LC3-II. DIDS, an anion-selective inhibitor, suppressed VacA-dependent increase in Cx43, suggesting that VacA channel activity was involved in this pathway. By confocal microscopy, Cx43 increased by VacA was predominately localized in cholesterol-rich, detergent-resistant membranes including GJs, and a fraction of Cx43 was incorporated in endocytotic vesicles and autophagolysosomes. Accumulation of Cx43 was also observed in gastric mucosa from *H. pylori*-infected patients compared with healthy controls, suggesting that the pathogen caused a similar effect *in vivo*. Our findings show that VacA-mediated effects on autophagy inhibits turnover of Cx43, resulting in increased levels in the cytoplasm, leading eventually to apoptotic cell death.

## Introduction

*Helicobacter pylori* (*H. pylori*) is a gram-negative bacterium that causes persistent infection in the gastric mucosa, with associated inflammation, gastric and duodenal ulceration, MALT lymphoma, and gastric cancer.^1^ *H. pylori* produces a number of virulence factors that are involved in pathogenesis of disease.^2,3^ Among them, vacuolating cytotoxin, VacA, is an exotoxin of about 90 kDa, which has been shown to be associated with gastric inflammation and ulceration in animal models of disease.^4^ VacA has two functional domains (p33–37 and p55–58). The p33–37 domain contributes to cytotoxicity and the p55–58 domain is responsible for binding to target cell receptors, for example, sphingomyelin, fibronectin, receptor protein-tyrosine phosphatase *α* (RPTP*α*), RPTP*β*, low-density lipoprotein receptor-related protein 1 (LRP1).^3,5^ Numerous studies have shown that VacA disrupts cell functions (e.g., autophagy, channel formation, intracellular vesicle traffic antigen presentation), leading eventually to cell death.^3,6^

Connexin 43 (Cx43), a member of the human Cx family, which is ubiquitously expressed and the most intensively studied of the Cx family,^7^ is responsible for VacA-induced cell death; its protein level is associated with sensitivity to VacA.^8^ In general, Cxs are integral membrane proteins, which form channels at gap junctions (GJs).^9,10^ They regulate intercellular communication, cell–cell channel formation and exchange of signaling molecules. This intercellular communication has a critical role in development and homeostasis.^10,11^ The amino-acid sequence and length of the C-terminal domain of Cx43 is unique compared with other Cxs.^12^ This C-terminal region has multiple modification sites (e.g., phosphorylation, ubiquitination, SUMOylation)^7^ and contributes to interaction with a number of functional proteins in GJs during pH-dependent channel closure.^13,14^ In contrast, the N-terminal domains of Cxs are involved in transjunctional voltage-dependent gating of GJ channels^15^ and oligomerization.^16^ Mutations in the N-terminal domain of Cx43 inhibit functional channel formation, and are found in a human developmental disorder known as oculodentodigital dysplasia.^14^ Furthermore, abnormal upregulation of Cx43 has been observed in diseased tissues (e.g., diabetic skin,^17^ blood vessels in proximity to a site of injury,^18^ arthritis,^19^ breast cancer and melanoma.^20^ Cx43-mediated cell–cell communication in GJs has an essential role in peritoneal metastasis.^21^ Interestingly, Cxs turnover is faster than that of the other transmembrane proteins, for example, the half-life of Cx43 in cultured cells is 1–5 h and is regulated by pathways involving proteasomes, lysosomes and autophagy.^22–24^

Autophagy is an important intracellular degradation system for maintaining cellular homeostasis.^25^ VacA-induced autophagy requires its channel-forming activity and induction of reactive oxygen species (ROS).^26,27^ We previously identified LRP1 as a receptor mediating VacA-induced autophagy, followed by apoptosis.^5^ In addition, Tsugawa *et al.* reported that *H. pylori* CagA protein, which is a type IV secretion effector and associated with the development of gastric cancer, was decreased by VacA-induced autophagy. Binding of VacA to LRP1 in AGS cells resulted in production of ROS and induction of autophagy, leading to CagA degradation.^5,28^ Interestingly, in some cells, Cx43 contributes to induction of autophagy^24,29^ and apoptosis.^30^ However, it is not clear whether Cx43 is associated with VacA-induced apoptosis and autophagy.

In the current study, we assessed the role of Cx43 in VacA-induced AZ-521 cell death and its presence in *H. pylori*-infected gastric mucosa. VacA did not affect Cx43 mRNA expression in AZ-521 cells, however, Cx43 accumulated in cytoplasm through slowed turnover by dysfunctional autophagy, leading eventually to apoptotic cell death in a glutathione (GSH)- and Rac1/ERK-dependent and LRP1-independent manner. Finally, consistent with our findings in cultured cells, we demonstrate increased Cx43 in the gastric mucosa of *H. pylori*-infected patients.

## Results

### VacA positively regulates Cx43 in AZ-521 cells

**Figure 1:**
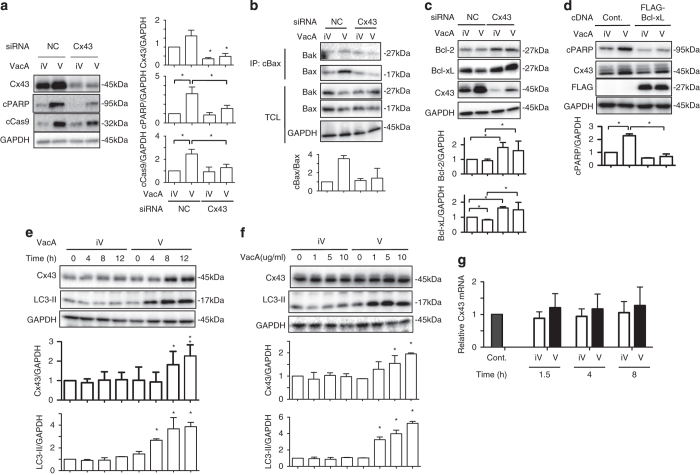
Increased Cx43 protein in VacA-treated AZ521 cells. (**a**) Control (NC) and Cx43 siRNA-transfected AZ-521 cells were incubated with 120 nM heat-inactivated (iV) or wild-type VacA (V) for 8 h, lysed with 1× SDS sample buffer and analyzed by immunoblotting with the indicated antibodies. Quantification of VacA-induced Cx43, cPARP and cCas9 levels in AZ-521 cells was performed by densitometry (right panel). Data are presented as mean±S.D. of values from three experiments and significance is *P<0.05. Experiments were repeated three times with similar results. (**b**) The indicated siRNA-transfected cells were treated with 120 nM heat-inactivated (iV) or wild-type VacA (V) for 6 h, lysed with cell lysis buffer containing 2% CHAPS and immunoprecipitated with anti-conformational changed Bax-specific antibody (cBax) as described in Materials and Methods. Experiments were repeated three times with similar results. Quantification of VacA-induced cBax levels in AZ-521 cells was performed by densitometry (bottom panel). (**c**) The indicated siRNA-transfected AZ-521 cells were incubated with 120 nM heat-inactivated (iV) or wild-type VacA (V) for 8–10 h, lysed with cell lysis buffer containing 1% NP40 and analyzed by immunoblotting with the indicated antibodies. Quantification of VacA-induced Bcl-2 and Bcl-xL levels in AZ-521 cells was performed by densitometry (bottom panels). Data are presented as mean±S.D. of values from four experiments and significance is *P<0.05. Experiments were repeated three times with similar results. (**d**) Control (Cont.) or FLAG-tagged Bcl-xL plasmid-transfected cells incubated with 120 nM heat-inactivated (iV) or wild-type VacA (V) for 10 h, lysed with 1× SDS sample buffer and analyzed by immunoblotting with the indicated antibodies. Quantification of VacA-induced cPARP levels in AZ-521 cells was performed by densitometry (bottom panel). Data are presented as mean±S.D. of values from three experiments and significance is *P<0.05. Experiments were repeated three times with similar results. (**e**) AZ-521 cells were incubated with 120 nM heat-inactivated (iV) or wild-type VacA (V) for the indicated times, lysed with 1× SDS sample buffer and analyzed by immunoblotting with the indicated antibodies. Quantification of VacA-induced Cx43 levels in AZ-521 cells was performed by densitometry (bottom panel). Data are presented as mean±S.D. of values from three experiments and significance is *P<0.05. Experiments were repeated three times with similar results. (**f**) AZ-521 cells were incubated with the indicated concentration of heat-inactivated (iV) or wild-type VacA (V) for 10 h, lysed with 1× SDS sample buffer and analyzed by immunoblotting with the indicated antibodies. Quantification of VacA-induced Cx43 levels in AZ-521 cells was performed by densitometry (bottom panel). Data are presented as mean±S.D. of values from three experiments and significance is *P<0.05. Experiments were repeated three times with similar results. (**g**) AZ-521 cells were incubated with 120 nM heat-inactivated (iV) or wild-type VacA (V) for the indicated times and total RNA was extracted as described in Materials and Methods. Cx43 mRNA was measured by real-time qPCR. Data are shown as mean±S.D. of values from three experiments. Results are shown as fold increase of GAPDH as an internal control. Experiments were repeated three times with similar results.

Recently, Radin *et al.* ^8^ showed that the expression level of Cx43 in cells contributes to VacA-induced cell death. To confirm this finding, we investigated the effect of Cx43 knockdown on VacA-induced apoptosis (Figure 1a). After incubation with VacA for 8 h, we found increased Cx43, associated with cleavage of caspase-9 (cCas9) and PARP (cPARP). In contrast, enhanced caspase-9 and PARP cleavage by VacA was significantly suppressed in Cx43-knockdown cells. In agreement, VacA induced Bax conformational changes in control cells, but not in Cx43-knockdown cells (Figure 1b). Previous studies reported that Cx43-mediated apoptosis is regulated by Bcl-2.^31,32^ We found that the basal expression levels of apoptosis blocker, Bcl-2 and Bcl-xL, were significantly increased in Cx43-knockdown cells compared with control cells (Figure 1c). In addition, VacA-induced PARP cleavage was suppressed in FLAG-tagged Bcl-xL-expressing cells, but did not affect VacA-dependent increase in Cx43 (Figure 1d). Induction of Cx43 in VacA-treated cells was observed after an 8 h incubation, whereas LC3-II generation, which is an index of autophagy and preceded apoptosis in VacA-treated cells,^5^ occurred in a 4 h incubation (Figure 1e). Induction of both Cx43 and LC3-II in VacA-treated cells were concentration dependent. Cx43 induction was significant at VacA concentrations >5 *μ*g/ml VacA (Figure 1f). Next, to assess whether VacA enhanced Cx43 transcription, we determined Cx43mRNA production by real-time qPCR (Figure 1g). The levels of Cx43 mRNA were not altered by VacA after 8 h incubation, suggesting that VacA may increase Cx43 accumulation by inhibiting its degradation, rather than by affecting its translation.

### Phosphorylation and ubiquitination of the increased cytoplasmic Cx43 observed in the presence of VacA

**Figure 2:**
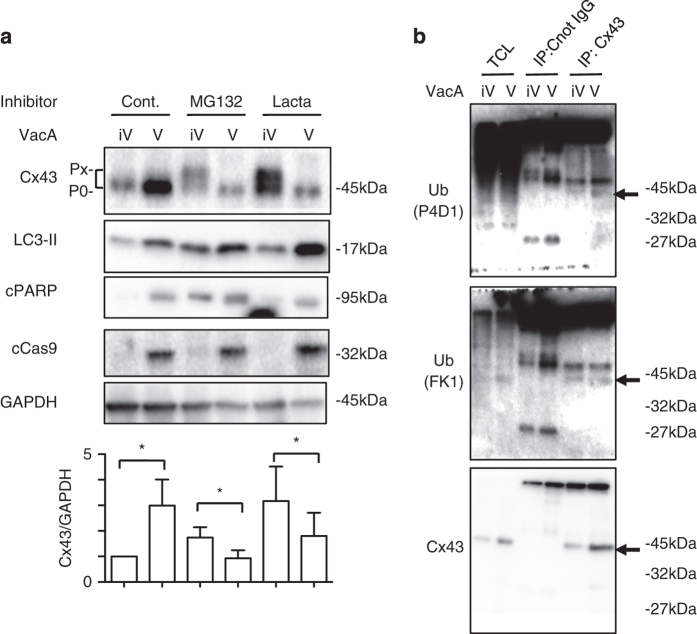
Increase of ubiquitinated Cx43 by VacA. (**a**) Cells were treated with 10 *μ*M MG132, 10 *μ*M lactacystin for 30 min, followed by incubation with heat-inactivated (iV) or wild-type VacA (V) for 10 h and subjected to immunoblots using the indicated antibodies. A blot representative of three separate experiments is shown. Quantification of VacA-induced Cx43 levels in AZ-521 cells was performed by densitometry (bottom panel). Data are presented as mean±S.D. of values from three experiments and significance is *P<0.05. (**b**) Cells were treated with 120 nM heat-inactivated (iV) or wild-type VacA (V) for 10 h, lysed with RIPA buffer, immunoprecipitated with anti-Cx43 antibodies and subjected to immunoblotting using anti-ubiquitin FK1 or P4D1 antibodies and anti-Cx43 antibody as described in Materials and Methods. Experiments were repeated two times with similar results.

It is well known that Cxs turnover is faster than that of other transmembrane proteins and its degradation is carried out by proteasome, endosome/lysosome and autophagy.^22–24^ As phosphorylation of Cx43 facilitated its degradation by the proteasome, we investigated whether proteasome inhibition affected the VacA-induced Cx43 increase. AZ-521 cells were treated with the proteasome inhibitors, MG132 and lactacystin, for 30 min followed by co-incubation with or without VacA (Figure 2a). VacA increased the Cx43-P_0_ band (non-phosphorylated Cx43) in control cells, whereas the Cx43-P_X_ band (phosphorylated Cx43) was detectable only when cells were treated with proteasome inhibitors. Proteasome inhibition blocked the degradation of Cx43-P_X_ in control cells. In VacA-treated cells, proteasome inhibitors did not block the degradation of Cx43-P_X_ and Cx43-P_0_ maintained its basal level in the presence of proteasome inhibitors. Thus, VacA did not stimulate the phosphorylation of Cx43, which is involved in regulation of GJ turnover and function. Proteasome inhibitors did not alter VacA-induced cPARP and cCas9, suggesting that phosphorylated Cx43 does not affect VacA-induced apoptosis. We next investigated if Cx43 was modified by ubiquitin. After cells were incubated with or without VacA, cell lysates were immunoprecipitated with anti-Cx43 antibody, and then analyzed with anti-ubiquitin antibodies (FK1 and P4D1). The P4D1 antibody recognizes all forms of ubiquitin, whereas FK1 antibody recognizes polyubiquitin chains.^33^ As shown in Figure 2b, VacA increased ubiquitinated Cx43, which is sorted through the endosomal or autophagic pathway, moving then to lysosomes where Cx43 is degraded. Our findings suggested that proteasomes did not affect autophagy and apoptosis via Cx43 in VacA-treated cells.

### Localization of VacA-increased Cx43 in autophagic vesicles, but not in mitochondria

**Figure 3:**
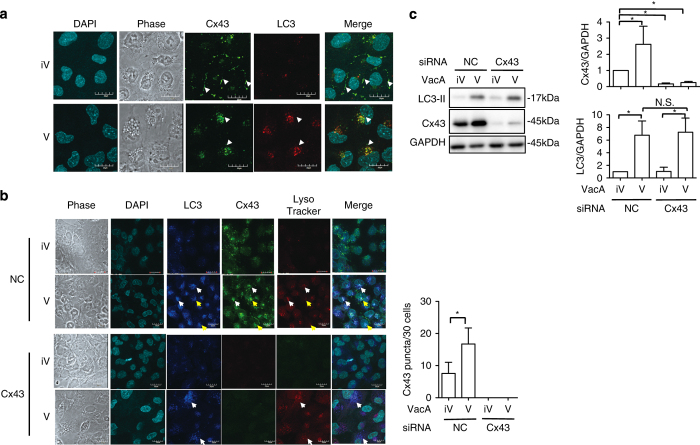

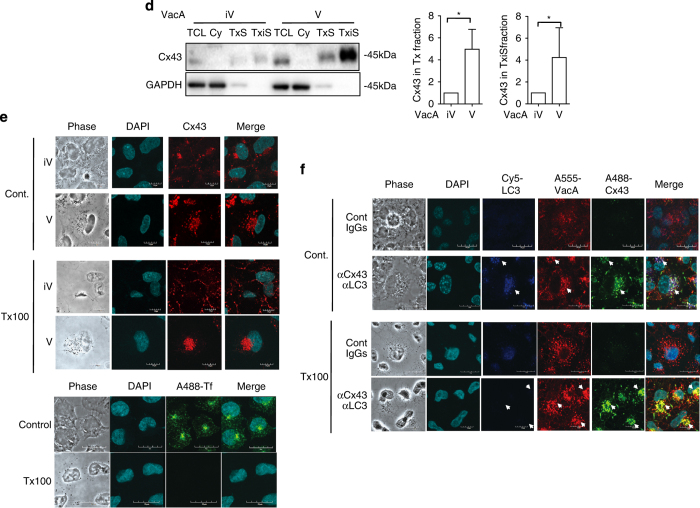

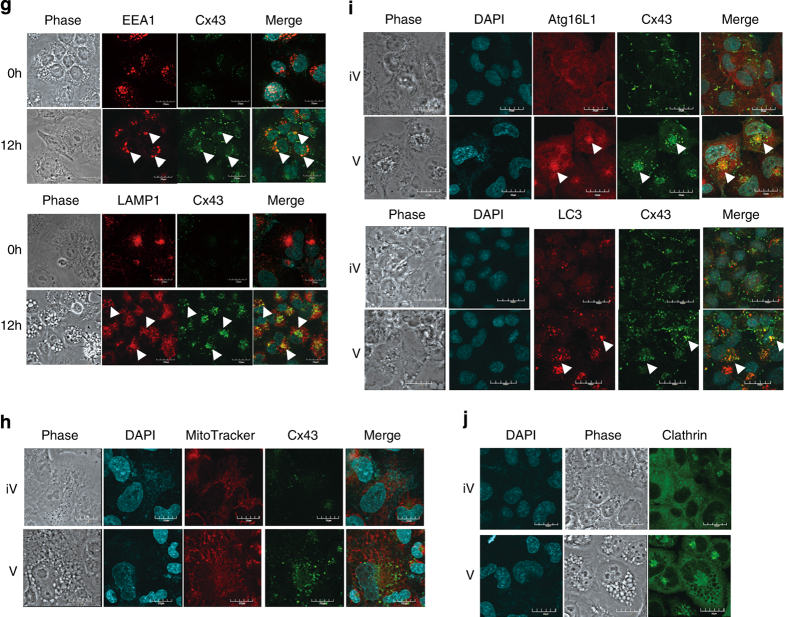
VacA-increased Cx43 colocalized with EEA1, LAMP1, Atg16L1 and LC3 in Tx-insoluble compartment. (**a**) AZ-521 cells were incubated with 120 nM heat-inactivated (iV) or wild-type VacA (V) for 10 h, then reacted with anti-Cx43 antibodies (green) and with DAPI (cyan). The arrows indicate Cx43 in plasma membranes. Bars represent 20 *μ*m. Experiments were repeated three times with similar results. (**b**) AZ-521 cells were incubated with 120 nM heat-inactivated (iV) or wild-type VacA (V) for 10 h and 100 nM LysoTracker (red) added to cells before fixation as described in Materials and Methods. Cells were reacted with anti-Cx43 antibodies (green) and DAPI (cyan). Merged and higher magnification images of the outlined areas are shown. Bars represent 20 *μ*m. Experiments were repeated two times with similar results. The arrows indicate the colocalization of LC3, Cx43 and LysoTracker. The number of Cx43 puncta in a single cell was manually counted under a confocal microscopy (right panel; *P<0.05). For each group, 30 cells were randomly selected for the average of number of Cx43 puncta in the cell. (**c**) The indicated siRNA-transfected cells were incubated with 120 nM heat-inactivated (iV) or wild-type VacA (V) for 10 h. Cells were lysed with 1× SDS sample buffer and analyzed by immunoblotting with the indicated antibodies. Quantification of VacA-induced Cx43 and LC3-II generation in AZ-521 cells was performed by densitometry (right panel). Data are presented as mean±S.D. of values from three experiments and significance is *P<0.05. Experiments were repeated three times with similar results. (**d**) After cells treated with heat-inactivated (iV) or wild-type VacA (V) for 10 h, cells were fractionated as described in Materials and Methods. TCL, total cell lysate; Cy, cytoplasm and small organelles; TxS, Triton X-100 soluble fraction. TxiS, Triton X-100 insoluble fraction. Proteins were applied to SDS-PAGE, followed by immunoblotting with the indicated antibodies. Quantification of VacA-induced Cx43 levels in Tx-soluble and TxiS-soluble fraction was performed by densitometry (bottom panel). Data are presented as mean±S.D. of values from three experiments and significance is *P<0.05. Experiments were repeated three times with similar results. (**e**) AZ-521 cells were incubated with 120 nM heat-inactivated (iV) or wild-type VacA (V) for 10 h and were treated with or without cold 1% Tx, fixed with 4% PFA, and reacted with anti-Cx43 antibodies (red) and incubated with DAPI (cyan) as described in Materials and Methods. Alexa488-labeled Transferrin was used as a positive control. Bars represent 20 *μ*m. Experiments were repeated three times with similar results. (**f**) AZ-521 cells were incubated with Alexa555-labeled VacA for 10 h and then were treated with or without cold 1% T×100, fixed with 4% PFA, and reacted with anti-Cx43 antibodies (green), anti-LC3 antibodies (blue) and with incubated DAPI (cyan). Bars represent 20 *μ*m. Experiments were repeated three times with similar results. The arrows indicate the colocalization of VacA, Cx43 and LC3. (**g**) AZ-521 cells were incubated with 120 nM heat-inactivated (iV) or wild-type VacA (V) for 10 h and then reacted with anti-EEA1 or anti-LAMP1 (red), or anti-Cx43 antibodies as indicated (green) and incubated with DAPI. Bars represent 20 *μ*m. Experiments were repeated three times with similar results. The arrows indicate the colocalization of Cx43 and EEA1 or LAMP1. (**h**) AZ-521 cells were incubated with 120 nM heat-inactivated (iV) or wild-type VacA (V) for 10 h; 50 nM MitoTracker (red) was added to cells before fixation as described in Materials and Methods. Cells were reacted with anti-Cx43 antibodies (green) and incubated with DAPI (cyan). Merged and higher magnification images of the outlined areas are shown. Bars represent 20 *μ*m. Experiments were repeated two times with similar results. (**i**) AZ-521 cells were incubated with 120 nM heat-inactivated (iV) or wild-type VacA (V) for 10 h, then reacted with anti-Atg16L1 antibodies (red), and anti-Cx43 antibodies (green) and incubated with DAPI. Bars represent 20 *μ*m. Experiments were repeated three times with similar results. The arrows indicate the colocalization with Cx43 and Atg16L1. (**j**) AZ-521 cells were incubated with 120 nM heat-inactivated (iV) or wild-type VacA (V) for 10 h. Cells reacted with anti-clathrin antibodies (green) and incubated with DAPI (cyan). Bars represent 20 *μ*m. Experiments were repeated two times with similar results.

By confocal microscopy, Cx43 localized at GJs in plasma membranes of control cells, while Cx43 accumulated in cytoplasmic compartments and colocalized with autophagosomal marker LC3 in VacA-treated cells (Figure 3a), suggesting that VacA-induced autophagy may be critical for apoptosis via Cx43. We also observed many puncta of LC3 and Cx43 and LysoTracker-positive vesicles in VacA-treated cells. All Cx43 colocalized with LC3 (white arrows), while some puncta of Cx43 with LC3 were not observed in autophagolysosome stained with LysoTracker, as shown by yellow arrows (Figure 3b). Silencing of the Cx43 gene with siRNA resulted, as expected, in interference with VacA-increased Cx43. On the basis of analysis of LC3-II generation by western blotting, however, generation of LC3-II and LysoTracker-positive vesicles was not inhibited by Cx43 knockdown, (Figure 3c), suggesting that there is another pathway that mediates VacA-induced autophagic vesicles independent of Cx43. VacA is degraded by autophagy after binding to LRP1, but not RPTPs, on the cell surface.^5^ Consistent with these findings, inhibition of LRP1 expression with LRP1 siRNA did not affect VacA-induced increase in cytoplasmic Cx43. Treatment of starved cells with an autophagy inhibitor, such as 3-methyladenine or chloroquine, increased the amount of Cx43 in GJs,^34^ which was resistant to extraction with Tx (Tx-insoluble fraction), indicating that blocking autophagy specifically prevented the loss of Cx43 at the plasma membrane. Cx43 was detected in both Tx-insoluble and Tx-soluble fractions of control cells, whereas VacA-increased Cx43 was enriched in Tx-insoluble fraction (Figure 3d). In agreement, confocal microscopic analysis showed that both VacA and VacA-increased Cx43 in cytoplasm were resistant to Tx extraction, whereas only Cx43 in GJs of control cells was resistant to this detergent (Figure 3e). By monitoring Alexa488-labeled transferrin, which is internalized by dynamin-dependent endocytosis,^35^ we found that Alexa488-labeled transferrin was completely extracted by Tx. In addition, we assessed whether VacA-induced Cx43 was colocalized with LC3 and VacA in these Tx-extraction-resistant compartments. As shown in Figure 3f, Cx43 colocalized with both LC3 and VacA in control cells. After treatment with T×100 buffer, most LC3 was lost, however, the vesicles containing both VacA and Cx43 were resistant to T×100 extraction. These data suggest that Cx43 and VacA were in vesicles containing cholesterol-rich membranes, which are resistant to Tx extraction.^36^ Taken together, Cx43, increased by VacA, localized in cytoplasmic vesicles, which consist of cholesterol-rich membranes as was found in GJs. Cx43, which localized in the mitochondria, influenced mitochondrial integrity and initiation of apoptosis.^37,38^ Cx43 also colocalized with autophagosome markers such as LC3^39^ and ATG16L1.^27^ By confocal microscopy, Cx43 increased by VacA colocalized with EEA1, LAMP1, Atg16L1 and LC3, suggesting that Cx43 is associated with cellular trafficking pathways involving endosomes and autophagy. However, VacA-increased Cx43 did not localize in mitochondria, which were detected by MitoTracker staining (Figures 3g–j).

### Atg16L1 and ROS/ERK signaling pathway participates in VacA-induced Cx43 increase

**Figure 4:**
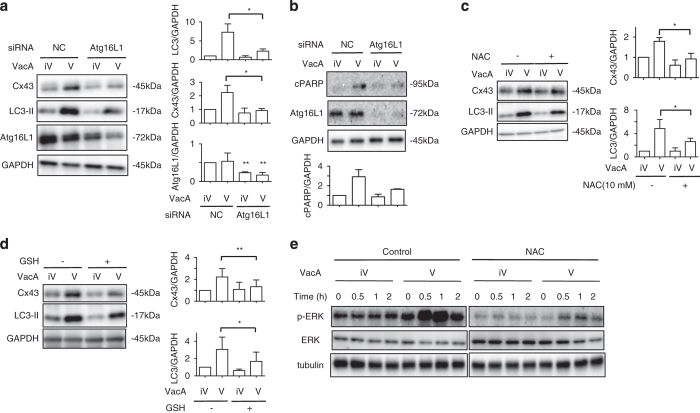

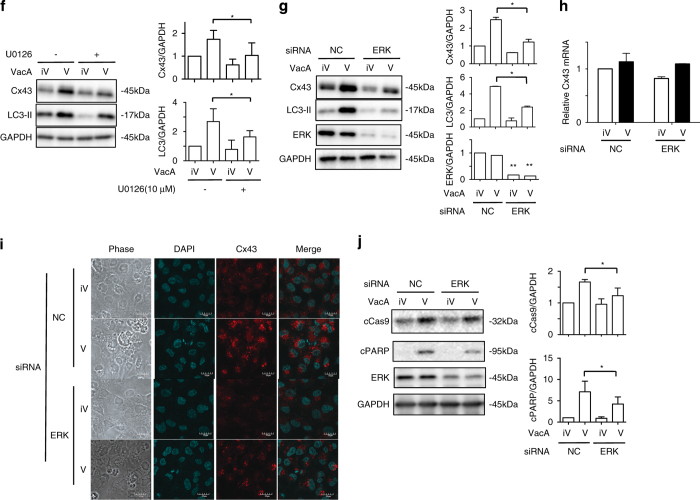
Atg16L1, ROS and ERK regulate Cx43 in the presence of VacA. (**a**) Control (NC) or Atg16L1 siRNA-transfected cells were incubated with 120 nM heat-inactivated (iV) or wild-type VacA (V) for 10 h. Cells were lysed with 1× SDS sample buffer and analyzed by immunoblotting with the indicated antibodies. Quantification of VacA-induced Cx43, LC3-II generation and Atg16L1 in AZ-521 cells was performed by densitometry (right panel). Data are presented as mean±S.D. of values from three experiments and significance is *P<0.05, **P<0.01. Experiments were repeated three times with similar results. (**b**) The indicated siRNA-transfected cells were treated with toxin as shown above. Cells were lysed with 1× SDS sample buffer and analyzed by immunoblotting with the indicated antibodies. Quantification of VacA-induced cPARP, Atg16L1 and GAPDH in AZ-521 cells was performed by densitometry (bottom panel). Data are presented as mean±S.D. of values from two experiments. Experiments were repeated two times with similar results. (**c** and **d**) AZ-521 cells were pretreated with or without 10 mM NAC (left panel) or 10 mM GSH (right panel) for 30 min and then incubated with 120 nM heat-inactivated (iV) or wild-type VacA (V) for 10 h. Cells were lysed with 1× SDS sample buffer and analyzed by immunoblotting with the indicated antibodies. Quantification of VacA-induced Cx43 and LC3-II generation in AZ-521 cells was performed by densitometry. Data are presented as mean±S.D. of values from three experiments and significance is *P<0.05 and **P<0.02. Experiments were repeated three times with similar results. (**e**) AZ-521 cells were pretreated with control (DMSO) or 10 mM NAC and then 120 nM heat-inactivated (iV) or wild-type VacA (V) for the indicated times. Cells were lysed with 1× SDS sample buffer and analyzed by immunoblotting with the indicated antibodies. Experiments were repeated three times with similar results. (**f**) AZ-521 cells were pretreated with 10 *μ*M U0126 for 30 min and then incubated with 120 nM heat-inactivated (iV) or wild-type VacA (V) for 10 h. Cells were lysed with 1× SDS sample buffer and analyzed by immunoblotting with the indicated antibodies. Quantification of VacA-induced Cx43 and LC3-II generation in AZ-521 cells was performed by densitometry (right panel). Data are presented as mean±S.D. of values from three experiments and significance is *P<0.05. Experiments were repeated three times with similar results. (**g**) Control (NC) or ERK siRNA-transfected cells were incubated with 120 nM heat-inactivated (iV) or wild-type VacA (V) for 10 h and lysed with 1× SDS sample buffer for immunoblotting with the indicated antibodies. Quantification of VacA-induced Cx43 and LC3-II generation and ERK levels in AZ-521 cells was performed by densitometry (right panel). Data are presented as mean±S.D. of values from three experiments and significance is *P<0.05, **P<0.01. Experiments were repeated three times with similar results. (**h**) The indicated siRNA-transfected cells were incubated with 120 nM heat-inactivated (iV) or wild-type VacA (V) for 4 h and total RNA was extracted as described in Materials and Methods. Cx43 mRNA was measured by real-time qPCR. Data are shown as mean±S.D. of values from two experiments. Results are shown as fold increase of GAPDH as an internal control. Experiments were repeated two times with similar results. (**i**) The indicated siRNA-transfected cells were incubated with 120 nM heat-inactivated (iV) or wild-type VacA (V) for 10 h and then reacted with anti-Cx43 antibodies (red) and incubated with DAPI (cyan). Bars represent 20 *μ*m. Experiments were repeated three times with similar results. (**j**) The indicated siRNA-transfected AZ-521 cells were incubated with 120 nM heat-inactivated (iV) or wild-type VacA (V) for 10 h and lysed with 1× SDS sample buffer for immunoblotting with the indicated antibodies. Quantification of VacA-induced cPARP and cCas9 was performed by densitometry (right panel). Data are presented as mean±S.D. of values from three experiments and significance is *P<0.05. Experiments were repeated three times with similar results.

We next examined whether autophagy regulates VacA-dependent increase in Cx43. VacA induced LC3-II generation independent of the increase in Cx43 (Figures 3b and c). Silencing of the Atg16L1 gene with Atg16L1 siRNA resulted in suppression of both Cx43 increase and LC3-II generation in VacA-treated cells as compared with control siRNA-transfected cells (Figure 4a). Thus, our results indicate that Atg16L1, which has an essential role in autophagy,^40^ participated in VacA-induced Cx43 increase and LC3-II generation. In addition, depletion of Atg16L1 significantly suppressed VacA-induced PARP cleavage (Figure 4b).

ROS is a key factor in induction of autophagy^41^ and the increase of Cx43 in cultured cardiomyocytes.^42^ A prior study demonstrated that VacA-reduced GSH is a trigger to induce LC3-II generation in AGS cells.^28^ *N*-acetyl-cysteine (NAC) is an antioxidant and free radical scavenger that increases intracellular GSH.^43^ We assessed whether GSH regulates VacA-induced Cx43 increase in AZ-521 cells and found that VacA-induced Cx43 increase and LC3-II generation were significantly suppressed by incubation with 10 mM NAC (Figure 4c) and 10 mM GSH (Figure 4d).

MAPK also regulates autophagy.^44^ Previous studies showed that VacA activates the ERK/MAP kinase cascade.^45^ Next, we investigated the effect of NAC on VacA-induced ERK activity. In control cells, VacA transiently increased ERK phosphorylation after 0.5 h incubation, with a return to basal level after 2 h. VacA-induced ERK phosphorylation, however, was suppressed in NAC-treated cells (Figure 4e). Furthermore, cells incubated with the ERK inhibitor, U0126 and VacA showed a significant decrease in the levels of Cx43 and LC3-II compared with cells treated with VacA only (Figure 4f). The ERK inhibitor did not affect VacA-induced vacuolating activity (Supplementary Figure S1b). Furthermore, ERK knockdown inhibited VacA-induced Cx43 increase and LC3-II generation (Figure 4g). ERK knockdown, however, did not affect Cx43 mRNA level (Figure 4h). We also monitored by confocal microscopy the effect of ERK knockdown on VacA-increased Cx43. VacA increased accumulation of Cx43 in control cells, whereas VacA did not increase accumulation of Cx43 puncta in ERK-knockdown cells (Figure 4i). More interestingly, ERK knockdown significantly suppressed VacA-induced cleavages of PARP and caspase-9 (Figure 4j). These data indicated that GSH level controls ERK activation, and thereby regulates VacA-increased Cx43 and LC3-II generation as well as apoptosis.

### Rac1 signaling pathway regulates VacA-induced Cx43 increase

**Figure 5:**
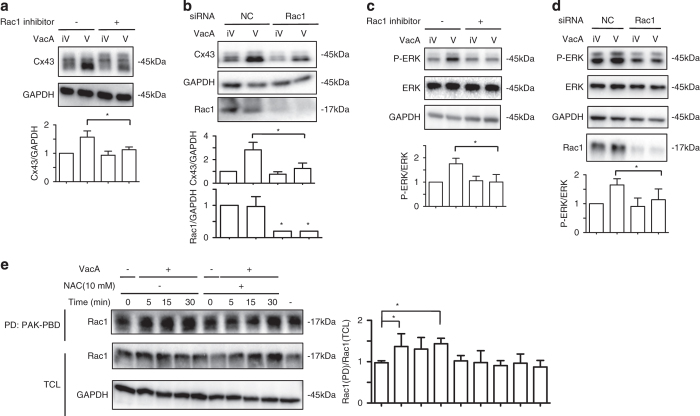
Rac1 involvement in VacA-increased Cx43. (**a**) AZ-521 cells were pretreated with 50 *μ*M Rac1 inhibitor, NSC27366, for 30 min and then incubated with 120 nM heat-inactivated (iV) or wild-type VacA (V) for 10 h. Cells were lysed with 1× SDS sample buffer and analyzed by immunoblotting with the indicated antibodies. Quantification of VacA-induced Cx43 in AZ-521 cells was performed by densitometry (bottom panel). Data are presented as mean±S.D. of values from three experiments and significance is *P<0.05. Experiments were repeated three times with similar results. (**b**) Control (NC) or Rac1 siRNA-transfected cells were incubated with 120 nM heat-inactivated (iV) or wild-type VacA (V) for 10 h and lysed with 1× SDS sample buffer and analyzed by immunoblotting with the indicated antibodies. Quantification of VacA-induced Cx43 in AZ-521 cells was performed by densitometry (bottom panel). Data are presented as mean±S.D. of values from four experiments and significance is *P<0.01. (**c**) Rac1 inhibitor-treated cells were incubated with 120 nM heat-inactivated (iV) or wild-type VacA (V) for 1 h and lysed with 1× SDS sample buffer and analyzed by immunoblotting with the indicated antibodies. Quantification of VacA-induced phospho-ERK (P-ERK) and ERK in AZ-521 cells was performed by densitometry (bottom panel). Data are presented as mean±S.D. of values from three experiments and significance is *P<0.01. (**d**) The indicated siRNA transfected cells were incubated with 120 nM heat-inactivated (iV) or wild-type VacA (V) for 1 h and lysed with 1× SDS sample buffer and analyzed by immunoblotting with the indicated antibodies. Quantification of VacA-induced phospho-ERK (P-ERK) and ERK in AZ-521 cells was performed by densitometry (bottom panel). Data are presented as mean±S.D. of values from three experiments and significance is *P<0.01. (**e**) AZ-521 cells were incubated with 120 nM wild-type VacA for 0, 5, 15, 30 min. After pull-down (PD) with PAK-PBD beads, active Rac1 was determined by immunoblotting with anti-Rac1 antibodies. The amounts of Rac1 and GAPDH in total cell lysate (TCL) were determined by immunoblotting with anti-Rac1 and anti-GAPDH antibodies. Data are presented as mean±S.D. of values from three experiments and significance is *P<0.05.

Hotchin *et al.* ^46^ showed that VacA activity is regulated by Rac1, a small GTP-binding protein that controls actin cytoskeleton reorganization and signal transduction. Furthermore, cytoskeletal reorganization is regulated by ROS, important mediators acting downstream of Rac1.^47^ We investigated here whether Rac1 is involved in the VacA-induced Cx43 increase. AZ-521 cells treated with the Rac1 inhibitor, NSC23766, in the presence of VacA, showed a significant decrease in the levels of Cx43 compared with treatment with VacA alone (Figure 5a). Furthermore, Rac1 knockdown inhibited VacA-induced Cx43 increase (Figure 5b). We further examined the effect of Rac1 on VacA-induced ERK phosphorylation. In the presence of the Rac1 inhibitor or following Rac1 knockdown, VacA-induced ERK phosphorylation was suppressed compared with treatment with VacA alone (Figures 5c and d). We next investigated whether VacA induced Rac1 activation. AZ-521 cells were incubated with VacA in the presence or absence of NAC. In control cells, VacA enhanced formation of Rac1-GTP in a time-dependent manner, which was suppressed in the presence of NAC (Figure 5e).

### Effects of anion-channel blocker and LRP1 knockdown on VacA-induced Cx43 increase

**Figure 6:**
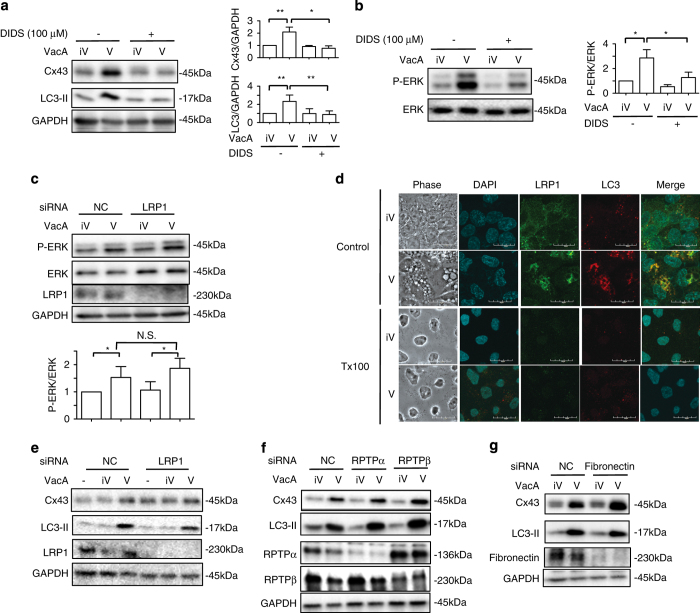
VacA channel activity, not LRP1, regulates VacA-induced Cx43 and LC3-II generation via ERK activation. (**a**) AZ-521 cells were pretreated with or without 100 *μ*M DIDS for 30 min and then incubated with 120 nM heat-inactivated (iV) or wild-type VacA (V) for 10 h. Cells were lysed with 1× SDS sample buffer and analyzed by immunoblotting with the indicated antibodies. Quantification of VacA-induced Cx43 and LC3-II generation in AZ-521 cells was performed by densitometry (right panel). Data are presented as mean±S.D. of values from three experiments and significance is *P<0.01, **P<0.05. Three independent experiments were performed with similar results. (**b**) AZ-521 cells were pretreated with control (DMSO) or 100 *μ*M DIDS and then 120 nM heat-inactivated (iV) or wild-type VacA (V) for 1 h at 37 °C. Cells were lysed with 1× SDS sample buffer for immunoblotting with p-ERK and ERK as a loading control. Quantification of p-ERK in AZ-521 cells was performed by densitometry (right panel). Data are presented as mean±S.D. of values from three experiments and significance is *P<0.05. Experiments were repeated independent three times. (**c**) Control (NC) or LRP1 siRNA-transfected cells were incubated with 120 nM heat-inactivated (iV) or wild-type VacA (V) for 1 h at 37 °C. Cells were lysed with 1× SDS sample buffer for immunoblotting with anti-ERK, anti-p-ERK and anti-LRP1 antibodies. GAPDH served as a loading control. Experiments were repeated three times with similar results. (**d**) AZ-521 cells were incubated with 120 nM heat-inactivated (iV) or wild-type VacA (V) for 10 h and were treated with or without cold 1% T×100, fixed with 4% PFA, and reacted with anti-LRP1 (green), anti-LC3 antibodies (red) and incubated with DAPI (cyan) as described in Materials and Methods. Bars represent 20 *μ*m. Experiments were repeated three times with similar results. (**e**–**g**) The indicated siRNA-transfected cells were incubated with 120 nM heat-inactivated (iV) or wild-type VacA (V) for 10 h at 37 °C. Cells were lysed with 1× SDS sample buffer and analyzed by immunoblotting with the indicated antibodies. GAPDH served as a loading control. Experiments were repeated three times with similar results.

Chloride channel blocker, DIDS, inhibited VacA-mediated channel activity and cellular vacuolation^48,49^ as well as LC3-II generation.^5^ We investigated the effect of 4,4′-disothiocyanatostibene-2,2′-disulfonic acid (DIDS) on VacA-induced Cx43 increase. DIDS significantly suppressed VacA-induced Cx43 increase as well as LC3-II generation (Figure 6a). In addition, VacA-induced ERK phosphorylation was also inhibited by DIDS (Figure 6b), suggesting that VacA-mediated channel activity triggered phosphorylation of ERK. VacA bound to specific receptors,^3,5^ which are involved in several pathological events. We investigated if LRP1 was the receptor associated with VacA-induced ERK phosphorylation. ERK phosphorylation by VacA was not affected by LRP1 knockdown (Figure 6c). As shown in Figure 4f, Cx43 and VacA were in vesicles containing detergent-resistant membranes (DRMs). We next investigated whether LRP1 and LC3-II also localized in DRMs. In control cells, VacA-induced LC3-II and internalized LRP1 were colocalized in cytoplasmic compartments as shown in a previous report.^5^ In contrast, both LRP1 and LC3-II were completely extracted by Tx, suggesting that LC3-II and LRP1 were found in cytoplasmic vesicles that did not include cholesterol-rich membranes (Figure 6d).

Although LRP1 knockdown suppressed LC3-II generation,^5^ VacA-induced Cx43 increase was not affected by LRP1 knockdown, indicating that LRP1 is not responsible for VacA-induced Cx43 increase (Figure 6e). Thus, ERK activation through LRP1 may be not associated with the signal transduction pathway responsible for VacA-induced Cx43 increase. Similarly, depletion of other VacA receptors such as RPTP*α*, RPTP*β* and fibronectin did not affect VacA-induced Cx43 increase and LC3-II generation (Figures 6f and g). These results raise the possibility that there might be a yet-to-be defined VacA receptor, which is responsible for the Cx43 increase.

### Increase of Cx43 in human biopsy samples in *H. pylori*-infected human gastric mucosa

**Figure 7:**
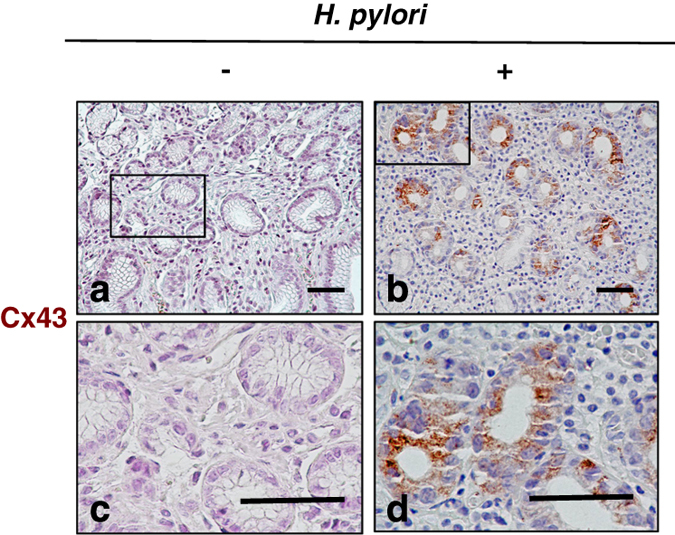
*H. pylori* infection is associated with increased Cx43 expression in human gut tissues. Cx43 was detected (i.e., brown staining) in *H. pylori-*negative (**a** and **c**) and -positive (**b** and **d**) gastric mucosa. Blue color indicated nuclear staining by hematoxylin. The black square shows low magnification views of the structures in **c** and **d**, respectively (**a** and **b**: ×20 ; **c** and **d**: ×80). The figure shows one experimental result representative of 5 *H. pylori*-negative and 11 of *H. pylori*-positive samples. Statistically significant difference between *H. pylori-*negative and -positive mucosa was observed (*P*=0.0256). Fisher’s exact test, *H. pylori*-positive *versus* -negative mucosa. Bars represent 50 *μ*m.

To assess the above findings in *H. pylori*-infected human mucosa, we investigated Cx43 protein level in the biopsy samples. The demographic characteristic of the patients is shown in Supplementary Table 1. Eleven *H. pylori*-positive and five *H. pylori-*negative biopsy samples from gastric antrum were examined. Interestingly, Cx43 content was not significantly increased in gastric mucosa of *H. pylori*-negative patients. In contrast, Cx43 was clearly observed in the gastric epithelium region in 8 out of 11 *H. pylori*-positive biopsy specimens (Figure 7), which was a statistically significant difference compared with *H. pylori*-negative mucosa (*P*=0.0256, Fisher’s exact test, *H. pylori*-positive *versus* -negative mucosa). These results suggested that Cx43 significantly accumulated in *H. pylori*-infected human gastric mucosa, with a potential role in the pathogenesis of human peptic diseases caused by *H. pylori.*

## Discussion

Cx43 is a ubiquitously expressed component of GJ channels and has an important role in intracellular communication involving cell death and cell survival.^10^ Cx43 overexpression in HeLa cells resulted in increased susceptibility to cell death caused by VacA. In addition, Cx43 knockdown by shRNA increased cellular resistance to VacA-induced cell death.^8^ Streptonigrin and *α*-Fas also enhanced cell death of Cx43-transfected HeLa cells compared with their wild-type counterparts.^30^ Our data show that incubation of AZ-521 cells with VacA for 4–12 h resulted in increased accumulation of Cx43 in the cytoplasm in association with apoptosis and autophagy. The accumulation of Cx43 occurred, even though Cx43 is rapidly degraded with a half-life of only a few hours.^50^ Consistent with a previous study,^8^ silencing of Cx43 gene reduced VacA-induced apoptosis, with increased basal levels of Bcl-2 and Bcl-xL, and suppressed Bak/Bax conformational changes, but did not affect vacuolating activity (Figures 1a–c and Supplementary Figure S1). Previous studies show that overexpression of Bcl-2 in Cx43-expressing cells suppressed apoptosis by chemotherapeutic agents.^31^ Indeed, we showed that VacA-induced PARP cleavage was significantly inhibited in Bcl-xL-expressing cells. Thus, our findings indicate that Cx43 is involved in VacA-induced apoptosis, which is regulated by Bcl-family proteins. The fact that we did not observe Cx43 in mitochondria stained with MitoTracker suggests that VacA did not increase mitochondrial Cx43, which is known to modulate apoptosis (Figure 3h). Furthermore, VacA treatment did not affect Cx43 mRNA content. These results are consistent with our hypothesis that VacA modulates Cx43 turnover through effects on its degradation, with Cx43 accumulation leading eventually to apoptotic cell death. On the other hand, Cx43 has cytoprotective effects against cell death, for example, loss of Cx43 expression led to astrocytic^51^ and osteocytic death.^52^ These diverse findings are consistent with the hypothesis that Cx43 signaling cascade might have different effects in different cell types.

**Figure 8:**
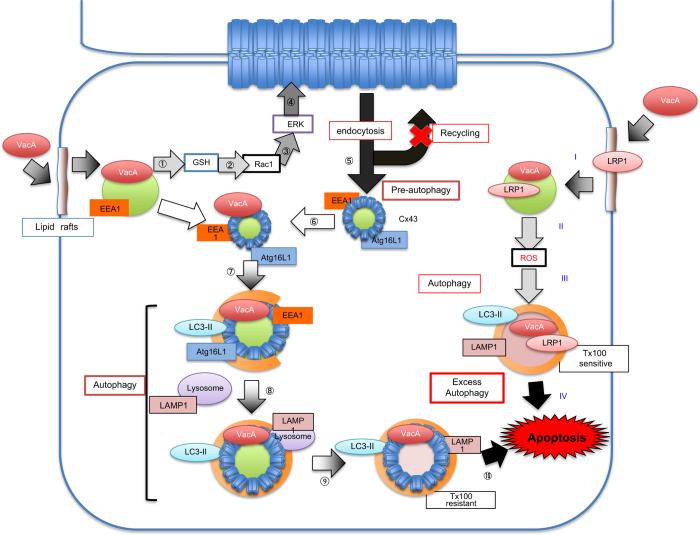
Proposed model of VacA effects on Cx43 and autophagy, followed by apoptosis. After VacA was translocated into cells via a lipid raft-dependent pathway, toxin channel activity is proposed to cause dysregulated GSH metabolism and activation of Rac1, followed by ERK phosphorylation (①–④). These signaling events promote Cx43 endocytosis. Cx43 enters endosome and pre-autophagy pathways as determined by the presence of several vesicle marker proteins (e.g., LC3, Atg16L1, EEA1 and LAMP1) (⑤–⑧). Cx43 accumulated in cytoplasmic compartments and colocalized with autophagosomal marker LC3 and VacA. The cytoplasmic compartments were predominantly localized in the Tx-insoluble fraction, suggesting its localization in cholesterol-rich DRMs (⑨–⑩). In contrast, LRP1-dependent endocytosis of VacA caused ROS-dependent autophagy and apoptosis (I–IV). These compartments were localized to the Tx-soluble fraction.

Beside regulation through differential gene transcription, it has been shown that Cx43 protein levels can be altered through lysosome- and proteasome-dependent degradation pathways. Confocal microscopic analysis showed that VacA causes the internalization of Cx43 from the plasma membrane, with reduction of membrane- localized Cx43, followed by accumulation of Cx43 in the cytoplasmic compartment, with several vesicle marker proteins (e.g., LC3, Atg16L1, EEA1 and LAMP1; Figures 3g and i). Of note, Cx43 knockdown did not affect VacA-induced LC3-II generation, whereas knockdown of both endosomal and autophagic marker proteins resulted in suppression of VacA-induced Cx43 increase in endosomal and autophagic vesicles. These data are consistent with the proposal that Cx43 was at least in part initially translocated by endocytosis to early endosomes, followed by translocation into autophagic vesicles, and then to lysosomes. Taken together, these results lead us to hypothesize that Cx43 turnover may be regulated through an endosome/autophagy pathway (Figure 8).

In addition, the VacA-induced increase in Cx43 was mainly localized in a Tx-insoluble fraction. Its cytoplasmic localization was in cholesterol-rich, DRMs, with a double-membrane structure as seen in GJ plaques^24^. Accumulation of Cx43 with VacA in DRMs seems to be due to the resistance to degradation by autophagy, followed by an increase in cytoplasmic Cx43, leading eventually to apoptosis. Indeed, a previous study showed that elevated Cx43 sensitizes cells to ER stress-induced, caspase-dependent apoptosis.^53^

Clathrin mediates both steps of internalization and recycling of Cx43 between GJ and early endosomes.^10^ Dynamin is essential for clathrin-dependent, coated-vesicle formation.^54^ We found that cells treated with dynasore, a dynamin inhibitor, showed inhibition of VacA-induced Cx43 increase although VacA still induced LC3-II accumulation (Supplementary Figure S2). VacA also caused the extraordinary clumping of clathrin (Figure 3j). These findings indicate that accumulation of cytoplasmic Cx43 by VacA may occur from the plasma membrane by a dynamin-dependent pathway. Thus, inhibition of clathrin-mediated turnover of Cx43 by VacA may cause the remodeling of Cx43-containing GJs.

We observed that VacA increased non-phosphorylated Cx43-P_0_ as shown in Figure 2a, consistent with a previous study,^55^ which showed that inhibition of proteasomal function by MG132 or lactacystin led to an increase in phosphorylated Cx43-P_x_, whereas VacA decreased Cx43-P_x_. These discrepant results suggest that, under control conditions, phosphorylated Cx43 is primarily eliminated by proteasomes. Kraft *et al.*^56^ reported that active crosstalk occurred between proteasome-mediated degradation and selective autophagy. However, in the presence of VacA, Cx43-P_x_ may be degraded by VacA-activated autophagolysosomes. Interestingly, VacA stabilized Cx43-P_0_ even in autophagolysosomes stained with LysoTracker Red, indicating that, in the presence of VacA, Cx43-P_0_ becomes resistant to autophagic degradation due to a change in its presence in cholesterol-rich membranes.

In cardiac myocytes, localization of Cx43 is regulated through the Rac1 pathway.^57^ A recent study suggested that Mena, a member of the Ena/VASP family of actin regulatory proteins, is controlled by Cx43 localization via regulation of Rac1.^58^ In addition, cytoskeleton reorganization is regulated by ROS, which act downstream of Rac1.^47^ These findings led us to investigate the possibility that regulation of Cx43 occurs downstream of Rac1. After confirming the effect of Rac1 inhibitor on VacA-induced vacuole formation^46^ (Supplementary Figure S1c), we showed that Rac1 inhibition and Rac1 knockdown significantly suppressed VacA-induced Cx43 increase (Figures 5a and b) and VacA-induced ERK phosphorylation (Figures 5c and d); these effects of VacA were not observed in Cdc42- and Rho-knockdown cells (Supplementary Figure S3). We next demonstrated that VacA-induced Rac1 activation was decreased in the presence of NAC (Figure 5e). These results indicate that VacA-induced Rac1 activation controls ERK activation, resulting in an increase in Cx43.

GSH acts as an antioxidant by directly interacting with ROS.^59,60^ Our previous study demonstrated that VacA suppressed the turnover rate of intracellular GSH, indicating that VacA impairs GSH metabolism in AZ-521 cells.^61^ In this study, we showed that VacA-induced Cx43 increase was significantly decreased by addition of GSH and NAC, suggesting that, in VacA-treated cells, disordered GSH metabolism triggers a Cx43 increase. In addition, it has been reported that the MAPK pathway is activated by depletion of GSH, followed by ROS generation.^62,63^ Furthermore, cultured cardiomyocytes treated with ROS showed an increase in Cx43 expression.^42^ Interleukin-1*β* increased Cx43 expression in synovial fibroblasts via an ERK-dependent pathway.^64^ In addition, a lipid-soluble pesticide, Lindane, activated ERK followed by induction of aberrant Cx43 endocytosis in 42GPA9 Sertoli cells.^65^ Despite our previous finding that LRP1 mediates VacA-induced LC3-II increase,^5^ LRP1 knockdown did not block VacA-induced ERK activation (Figure 6c), suggesting that there are at least two pathways, ERK-dependent and ERK-independent, to induce LC3-II generation by VacA and that ERK activation through LRP1 may not be responsible for VacA-induced Cx43 increase (Figure 6e). Thus, these findings suggest that VacA-induced Cx43 increase and LC3-II generation are associated with a ROS-dependent ERK signaling cascade.

*H. pylori* infection has an important role in pathogenesis of not only stomach or duodenal^66^ but also a variety of skin^67^ and lung diseases.^68^ Thus, it seems that *H. pylori* causes systemic disease. Abnormal upregulation of Cx43 has been observed in several diseases.^17–21^ Interestingly, reduction of Cx43 expression has been shown to be associated with enhanced wound closure.^69–71^

Our study demonstrated the elevated Cx43 in *H. pylori-*infected human gastric tissue, was associated with gastritis/erosion compared with healthy mucosa. It remains to be determined whether Cx43 has a role in the pathogenesis of disease due to *H. pylori* infection. However, most of the *H. pylori* isolated from Japanese gastric mucosa are VacA positive. Thus, VacA may participate in the generation of increased Cx43 *in vivo.* Interestingly, Liu *et al.*^72^ reported that Cx43 expression decreases as gastric mucosa progresses to a precancerous lesion and then to cancer. These data are in agreement with our model, as the expected rate of epithelial apoptosis would generally decline during carcinogenesis. It has also been shown that during *H. pylori*-associated gastric cancer, the Cx43 gene promoter was hyper methylated, resulting in decreased Cx43 mRNA expression.^73^ Our findings imply that VacA-increased Cx43 may contribute to tissue damage through epithelial apoptosis during *H. pylori* infection. Cx43 may thus be a potential therapeutic target. Reduction of Cx43 may have anti-inflammatory effects and inhibit the development of *H. pylori* VacA-induced tissue damage.

## Materials and Methods

### Antibodies and other reagents

Anti-LC3B, anti-Bcl-xL, anti-Atg16L1, anti-Rac1, anti-Rho1, anti-Cdc42, anti-phospho-ERK, anti-EEA1 and anti-LAMP1 antibodies were purchased from Cell Signaling Technology (Danvers, MA, USA). Mouse monoclonal antibodies reactive with LRP1 (11H4) were a kind gift from Dr. Strickland, University of Maryland School of Medicine, Baltimore. Fibronectin (EP5) and ubiquitin (P4D1) were from Santa Cruz Biotechnologies (Santa Cruz, CA, USA); anti-Cx43, anti-ERK, anti-Bcl-2 and anti-RPTP*β* antibodies were from BD Biosciences (Tokyo, Japan); anti-multi ubiquitin monoclonal antibody (FK1) was from MBL (Nagoya, Japan); anti-GAPDH antibody was from GeneTex (Irvine, CA, USA) and anti-LC3 (clone 1703) antibody was from Cosmo Bio (Tokyo, Japan). Anti-RPTP*α* rabbit polyclonal antibodies for immunoblotting were provided by Dr. Jan Sap; anti-*α*-tubulin and anti-FLAG M2 antibodies, *N*-acetyl-l-cysteine (NAC) and ammonium chloride were from Sigma Aldrich (St. Louis, MO, USA); ProLong Gold Antifade Reagent with DAPI and DIDS were from Invitrogen (Eugene, OR, USA); an ERK inhibitor, U0126, was from Cayman Chemical (Ann Arbor, MI, USA); Rac1 inhibitor, NSC23766 was from Wako Pure Chemical Industries (Osaka, Japan); and GSH was from Merck (Darmstadt, Germany).

### Cell culture and gene silencing

AZ-521 cells had been considered a gastric cancer cell line. However, RIKEN BioResource Center recently reported that AZ-521 is, in fact, a misidentified HuTu-80 cell line derived from human duodenum carcinoma (http://www.brc.riken.jp/lab/cell/english/urgent_AZ521.pdf). We confirmed by short tandem repeat analyses that AZ-521 cells are identical to HuTu-80 cells (Promega, Oosaka, Japan). AGS cells are a human gastric cancer cell line. Both cell lines were cultured in Earle's minimal essential medium (Sigma) containing 10% fetal calf serum. Cells were plated into 24-well (5×10^4^ cells per well) or 12-well dishes (2–3×10^5^ cells per well) in EMEM containing 10% FCS. ERK siRNA (5′-cuccaaagcucuggauuuatt-3′) and fibronectin siRNA (5′-cccgguuguuaugacaauggatt-3′) were synthesized by Sigma Aldrich. Atg16L siRNA (5′-caggacaatgtggatactcat-3′) were designed and validated as described.^74^ Rac1 siRNA (5′-caccacugucccaacacuctt-3′) was designed and validated as described.^75^ RPTP*β* siRNA and RPTP*α* siRNA were synthesized by B-Bridge, as described previously.^5^ Negative-control siRNAs were purchased from Sigma Aldrich. LRP1 siRNA was purchased from Ambion (Carlsbad, CA, USA). AZ-521 or AGS cells were transfected with 100 nM of the indicated siRNAs for 48–72 h using Lipofectamine RNAiMax transfection reagent (Invitrogen, Carlsbad, CA, USA) according to the manufacturer’s protocol. Knockdown of the target proteins was confirmed by immunoblotting with the indicated antibodies.

### Purification of VacA

The toxin-producing *H. pylori* strain ATCC 49503 was the source of VacA for purification as previously described.^76^

### Assay for vacuolating activity

Vacuolating activity was assessed using AZ-521 cells as previously described.^76^ Briefly, cells (1×10^4^ cells per well, 100 *μ*l) were grown as monolayers in 96-well culture plates for 24 h in a 5% CO_2_ atmosphere at 37 °C. VacA was added, and cells were incubated at 37 °C for the indicated times. To quantify vacuolating activity, uptake of neutral red into vacuoles was determined.

### Western blot analysis

AZ-521 cells were treated with heat-inactivated VacA or VacA for the indicated times at 37 °C. Cells were lysed with 100 *μ*l of SDS-sample buffer (62.5 mM Tris, pH 6.8, 1% SDS, 10% glycerol, 5 mM dithiothreitol, 0.001% bromophenol blue) or 150 *μ*l of cell lysis buffer (50 mM Tris-HCl, pH 7.7, 0.15 M NaCl, 1% NONIDET P-40 (NP40) and protease inhibitor cocktail (Roche, Mannheim, Germany)). After SDS-PAGE, proteins were transferred to Immobilon-P membranes (Millipore, Temecula, CA, USA), which were incubated with primary antibodies overnight at 4 °C, then washed with TTBS three times, incubated with the appropriate HRP-linked secondary antibodies at room temperature for 1 h, washed with TTBS three times, and finally incubated in Super Signal West Pico mixture (Thermo Scientific, Waltham, MA, USA). HRP-bound protein bands were visualized by Las-1000 (GE Healthcare Life Science, Buckinghamshire, UK).

### Construct of Bcl-xL expression plasmid and transfection

Total RNA was extracted from AZ-521 cells using ISOGEN II (NIPPON GENE, Tokyo, Japan) according to the manufacturer’s instructions. Complementary DNA (cDNA) was synthesized from 5 *μ*g of total RNA using Primer Script II 1st strand cDNA Synthesis Kit (TaKaRa Bio, Shiga, Japan). Primers used for Bcl-xL amplification were 5′-gaattcgccaccatgtctcagagcaac;cgggagct-3′ and 5′-gtcgactttccgactgaagagtgagcccagcagaa-3′. cDNA was amplified in 25 *μ*l of PrimeSTAR Max mixture according to the manufacturer’s protocol (TaKaRa Bio). The PCR conditions were as follows: 35 cycles of 98 °C for 10 s, 55 °C for 15 s and 72 °C for 60 s. To add 3’ adenine-overhangs, ExTaq polymerase (0.5 *μ*l, TaKaRa Bio) was incubated with the reaction mixture at 72 °C for 10 min. PCR products were subjected to electrophoresis on 1% agarose gels containing ethidium bromide, and the band was extracted with a FastGene Gel/PCR Extraction Kit (Nippon Genetics Co. Ltd., Tokyo, Japan), subcloned into pMD20-T vector (TaKaRa Bio) and then inserted in the cloning sites (EcoR1 and Sal1) of pFLAG-CMV-5a vector (Sigma Aldrich). Cells were cultured in a 24-well plate (1×10^5^ cells per well) overnight and transfected with 0.5 *μ*g of plasmids using X-tremeGENE HP DNA Transfection Reagent (Roche). After a 24-h incubation, cells were treated with the toxins for 10 h.

### Immunofluorescence confocal microscopy

For immunofluorescence analysis of VacA colocalization with target proteins, AZ-521 cells (1×10^5^ cells) cultured on coverslips (Matsunami, Oosaka, Japan) were incubated with 120 nM VacA for the indicated time. Cells were fixed with 4% paraformaldehyde at room temperature for 15 min, washed with PBS twice, and then immediately permeabilized with ice-cold 100% methanol for 10 min at −20 °C. The cells are then rinsed three times with PBS and incubated with blocking buffer (5% goat serum, 0.3% Triton X-100 in PBS) at room temperature for 1 h. To visualize Cx43 (monoclonal, 1 : 200) and LC3B (polyclonal, 1 : 200), cells were further incubated with the primary antibodies in 1% BSA/ PBS buffer at 4 °C overnight, washed twice with PBS and incubated with anti-rabbit Alexa488 (Molecular Probes, Carlsbad, CA, USA), anti-mouse Alexa488 (Molecular Probes), or anti-mouse Cy5 (Jackson ImmunResearch Laboratories Inc., West Grove, PA, USA) antibodies at room temperature for 1 h in the dark. After washing with PBS three times, cells were mounted on glass slides using Prolong Gold Antifade reagent with DAPI. For staining the lysosomal and mitochondrial compartments, respectively, VacA-treated cells were incubated with 100 nM LysoTracker Red DND-99 (Molecular Probes) or 50 nM MitoTracker (Molecular Probes) according to the instruction manual, before fixation with 4% paraformaldehyde. Colocalization of VacA and the indicated proteins was analyzed by FV10i-LIV confocal microscopy (Olympus, Tokyo, Japan). The images were arranged with Adobe Photoshop CS4.

### Subcellular fractionation

Subcellular fractionation was performed according to a previous study.^77^ Briefly, AZ-521 cells (3×10^5^ cells per six-well plate) were disrupted by Dounce homogenization in 100 *μ*l of hypotonic buffer with 100 strokes, then centrifuged for 5 min at 800×*g* at 4 °C. The supernatant (total cell lysate fraction) was centrifuged for 15 min at 17 400×*g* at 4 °C. The supernatant (cytoplasmic fraction) was collected. The pellet was suspended in 50 *μ*l of cell lysis buffer (50 mM Tris-HCl, pH 7.7, 0.15 M NaCl, 10% glycerol, 1% Triton X-100 (Tx) and protease inhibitor cocktail (Roche)) and then incubated for 30 min on ice. After centrifugation for 15 min at 17 400×*g* at 4 °C, the supernatant (Tx-soluble fraction) was collected and the pellet was solubilized with 50 *μ*l of 1× SDS sample buffer (Tx-insoluble fraction).

### Triton extraction

Triton X-100 extraction of AZ-521 cells cultured on coverslips was performed as described previously.^78^ After incubation with Alexa555-labeled VacA, Alexa488-labeled transferrin, heat-inactivated VacA (iV) or VacA (V) for the indicated time at 37 °C, cells were cooled to 4 °C, incubated with cold 1% Tx in 50 mM Tris-HCl, 0.15 M NaCl pH7.7 for 20 min, then washed with cold PBS, fixed with 4% PFA and analyzed by confocal microscopy.

### Immunoprecipitation

Co-immunoprecipitation of conformationally changed Bax or Bak was performed as described previously.^79^ Briefly, the indicated siRNA-transfected AZ-521 cells were treated with heat-inactivated VacA (iV) or VacA (V) for 6 h. Cells were solubilized with cell lysis buffer (10 mM HEPES, 150 mM NaCl, 1.5 mM MgCl_2_, 1 mM EGTA, 2% CHAPS, pH 7.4), centrifuged at 17 400×*g* for 15 min at 4 °C, and incubated with conformation-specific anti-Bax antibody (clone 3) (BD Biosciences) or anti-Bak antibody (Ab-2) (Calbiochem, San Diego, CA, USA) at 4 °C for 3 h. Immunocomplexes were collected by incubation with protein G-Sepharose (Invitrogen), washed with cell lysis buffer three times, and dissolved in SDS-sample buffer. These samples were analyzed by SDS-PAGE in 15% gels, and transferred to PVDF membranes, which were then analyzed by immunoblotting using anti-Bax or anti-Bak antibodies (Cell Signaling Technology).

After incubation with heat-inactivated VacA (iV) or VacA (V) for 8–10 h, cells were solubilized with RIPA buffer, centrifuged at 17 400×*g* for 15 min at 4 °C, and then incubated with anti-Cx43 monoclonal antibody (BD) overnight at 4 °C. Immunocomplexes were washed with RIPA buffer three times, and dissolved in SDS-sample buffer. These samples were analyzed by SDS-PAGE in 15% gels, and transferred to PVDF membranes, which were then analyzed by immunoblotting using the indicated antibodies.

### Real-time quantitative PCR analysis

Total RNA from AZ-521 cells (2×10^5^ cells) was extracted by ISOGEN II (Wako) as described in the instruction manual. First-strand cDNA synthesis was performed with a PrimeScript II 1st strand cDNA Synthesis Kit (Takara Bio). Real-time quantitative PCR (qPCR) analysis was conducted with the fluorescent dye SYBR Green methodology using Power SYBR Green PCR Master Mix (ABI) and ABI Prism 7300 (Applied Biosystems Inc, Carlsbad, CA, USA). Primer pairs for the Cx43 and GAPDH genes were obtained from Takara. The PCR protocol was performed as described in the instruction manual: the reaction mix containing AmpliTaq Gold DNA polymerase was incubated for 10 min at 95 °C, followed by denaturation for 15 s at 95 °C with annealing and extension for 1 min at 60 °C, for 45 cycles. The dissociation curve for each sample was analyzed to verify the specificity of each reaction. The relative mRNA expression levels of Cx43 genes were determined by the delta-delta Ct method and normalized to GAPDH expression.

### Detection of Rac1 activation

To detect the active form of Rac1, we expressed and purified GST-PAK1-PBD (Addgene 12217), which was conjugated with Glutathione Sepharose 4B (GE Healthcare Life Science, Piscataway, NJ, USA). Cells were lysed with Cell Lysis Buffer (#CLB01, Cytoskeleton) including protease inhibitors, centrifuged at 17 200×*g* for 2 min at 4 °C, and then the supernatant was incubated with GSH-PAK1-PBD-conjugated beads for 2 h at 4 °C to pull-down the GTP-bound form of Rac1. Afterwards, the beads were washed once with cell washing buffer (#WB01, Cytoskeleton), and suspended in 4× SDS sample buffer. The GTP-bound form of Rac1was detected by immunoblotting using anti-Rac1 antibody (Cell Signaling Technology).

### Human biopsy samples

Patients who underwent upper gastrointestinal endoscopy from 2013 April to 2015 May for medical reasons were recruited to participate in the study. Samples were obtained in agreement with the Helsinki Declaration. The study was reviewed and approved by an Ethics Committee of Nagasaki University Hospital (Office of Human Subjects Protection Registration number IORG0007678). During endoscopic examination, biopsy specimens were obtained from the duodenum bulbus and gastric antrum along the lesser curvature. The specimen was fixed in 10% formalin and embedded in paraffin for histopathological examination. *H. pylori* infection was assessed by either the rapid urease test (Helicocheck, Otsuka Pharmaceutical, Tokushima, Japan) or histology with Giemsa staining. Patients were considered to have *H. pylori* infection when at least one of these examinations was positive. Patients were defined as *H. pylori* negative when all test results were negative.

### Immunohistochemistry

After antigen retrieval by commercially available solution (Target Retrieval Solution, DakoCytomation, DAKO, Glostrup, Denmark) at 95 °C for 20 min, sections were pre-incubated with 0.3% H_2_O_2_/methanol for 5 min. Tissues were then incubated with an anti-Connexin 43 rabbit polyclonal antibody (Cell Signaling Technology), at a 1 : 25 dilution in Dako Antibody Diluent with Background Reducing Components (DakoCytomation, DAKO), followed by reaction with secondary antibody in Dako EnVision System-HRP Labelled polymer Anti-Rabbit (DakoCytomation, DAKO). Then, the slides were colorized with chromogenic substrate DAB (DakoCytomation, DAKO) for 5 min and were counterstained with hematoxylin.

### Statistics

Densitometric analysis on the immunoblots was done by Image Gauge software (FUJIFILM). The *P-*values for densitometric analysis were determined by one-tailed Student’s *t-*test (unpaired) with Prism 5 (Graphpad, San Diego, CA, USA). Statistical analysis regarding the presence or absence of Cx43 expression in *H. pylori*-positive and -negative human gastric mucosa was assessed by Fisher’s exact test. *P*-values of <0.05 were considered statistically significant.

## Abbreviations

VacA: vacuolating cytotoxin
*H. pylori*: *Helicobacter pylori*
Cxs: connexins
Cx43: connexin 43
GJ: Gap junction
NAC: *N*-acetyl-cysteine
DRMs: detergent-resistant membranes
RPTP: receptor protein-tyrosine phosphatase
ROS: reactive oxygen species
LRP1: low-density lipoprotein receptor-related protein 1
PFA: paraformaldehyde
SDS-PAGE: sodium dodecyl sulfate-polyacrylamide gel electrophoresis
cCas9: cleaved caspase 9
PARP: poly(ADP-ribose)polymerase
cPARP: cleaved PARP
Tx: Triton X-100
NC: negative- control siRNA.
