# Supplementary figures and images for: Helicobacter pylori VacA induces apoptosis by accumulation of connexin 43 in autophagic vesicles via a Rac1/ERK-dependent pathway
Source: Cell Death Discov. 2015 Sep 28;1:15035–. doi: 10.1038/cddiscovery.2015.35 (PMC4979424; doi:10.1038/cddiscovery.2015.35)

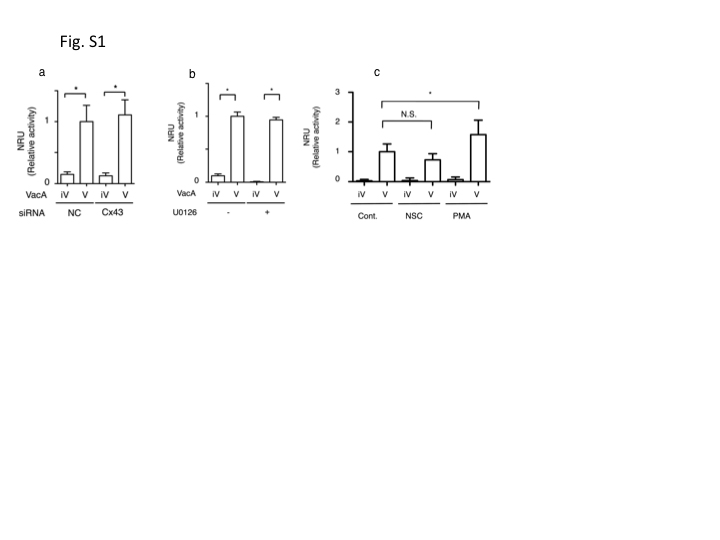

Supplement: Supplementary Figure S1 [file cddiscovery201535-s1.jpg]

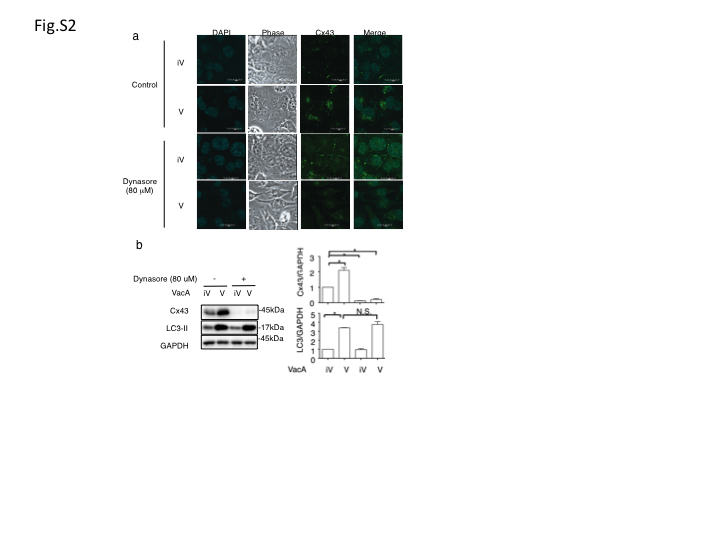

Supplement: Supplementary Figure S2 [file cddiscovery201535-s2.jpg]

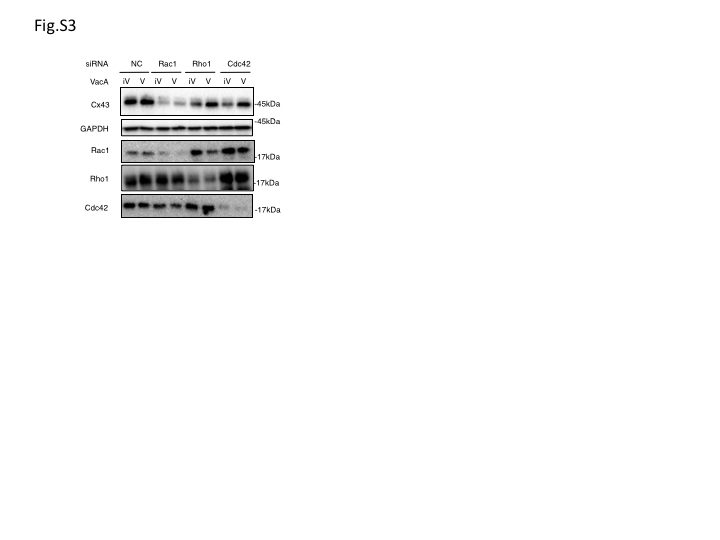

Supplement: Supplementary Figure S3 [file cddiscovery201535-s3.jpg]

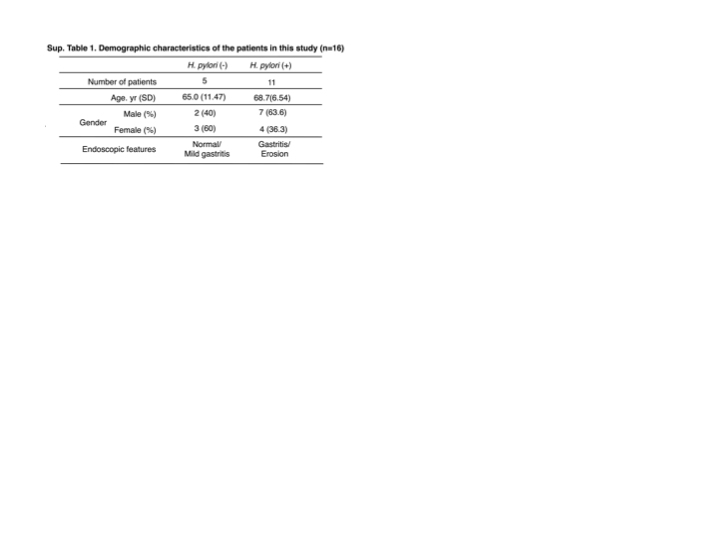

Supplement: Supplementary Table 1 [file cddiscovery201535-s4.jpg]
