# Supplementary material for: Helicobacter pylori VacA induces apoptosis by accumulation of connexin 43 in autophagic vesicles via a Rac1/ERK-dependent pathway
Source: Cell Death Discov. 2015 Sep 28;1:15035–. doi: 10.1038/cddiscovery.2015.35 (PMC4979424; doi:10.1038/cddiscovery.2015.35)
Supplement: Supplementary Figure and Table Legends [file cddiscovery201535-s5.doc]

**Supplementary Information**

**Figure S1. VacA-induced vacuolating activity in Cx43-knockdown cells and in U0126-, NSC23766- or PMA-treated cells.**

(a) After NC or Cx43 siRNA-transfected cells were incubated with 120 nM heat-inactivated (iV) or wild-type VacA (V) for 18 h. Vacuolating activity was assessed by neutral red uptake. (b and c) AZ-521 cells were incubated initially with 10 μM U0126, 50 μM NSC23766 or 100 ng/ml PMA and then incubated with 120 nM heat-inactivated (iV) or wild-type VacA (V) for 18 h. Vacuolating activity was assessed by neutral red uptake. Data are presented as mean ±SD and significance is **P*<0.05. Experiments were repeated three times with triplicates.

**Figure S2. Dynasore inhibited VacA-increased Cx43.**

1. AZ-521 cells were pretreated with or without 80 μM dynasore for 30 min and then incubated with 120 nM heat-inactivated (iV) or wild-type VacA (V) for 10 h. Cells were reacted with anti-Cx43 antibodies (green) and incubated with DAPI. Bars represent 20 μm. Experiments were repeated three times with similar results.
2. After cells were treated with Dynasore as described above, cells were lysed with 1xSDS sample buffer for immunoblotting with the indicated antibodies. Quantification of Cx43 and LC3-II in AZ-521 cells was performed by densitometry (right panel). Data are presented as mean ±SD of values from three experiments and significance is **P*<0.05. Experiments were repeated three times.

**Figure S3. Rho1 and Cdc42 were not involved in the increase in Cx43 by VacA.**

NC, Rac1, Rho1 or Cdc42 siRNA-transfected cells were incubated with 120 nM heat-inactivated (iV) or wild-type VacA (V) for 10 h and lysed with 1xSDS sample buffer and analyzed by immunoblotting with the indicated antibodies. Experiments were repeated two times with similar results.

**Supplemental Table 1. Demographic characteristics of the patients in this study.**
